# Ageing as key factor for distant metastasis patterns and prognosis in patients with extensive-stage Small Cell Lung Cancer

**DOI:** 10.7150/jca.49681

**Published:** 2021-01-15

**Authors:** Junyong Zou, Shijie Guo, Meng Ting Xiong, Yingchun Xu, Jiale Shao, Zhongkai Tong, Peng Zhang, Long Pan, Aimei Peng, Xuan Li

**Affiliations:** 1 Department of Respiratory Medicine, Shanghai 10th People's Hospital, Tongji University School of Medicine, Shanghai 200072, China.; 2 Department of Respiratory Medicine, Hwamei Hospital, University of Chinese Academy of Sciences, Ningbo 315010, China.; 3 Ningbo Institute of Life and Health Industry, University of Chinese Academy of Sciences,Ningbo 315010, China.; 4 Department of Neurology, Shanghai Tenth People's Hospital, Tongji University School of Medicine, Shanghai 200072, China.; 5 Department of Tuberculosis, Pulmonary Hospital, Tongji University School of Medicine, Shanghai 200072, China.; 6 Tongji University School of Medicine, Shanghai 200092, China.; 7 Department of Cardio-Thoracic Surgery, Shanghai Tenth People's Hospital, Tongji University School of Medicine, Shanghai200072, China.; 8 Department of Interventional and Vascular Surgery, Shanghai Tenth People's Hospital, Tongji University School of Medicine, Shanghai200072, China.

**Keywords:** age-related, small cell lung cancer, metastasis, survival, prognosis

## Abstract

**Background:** Small cell lung cancer (SCLC) represents about 13% of lung cancer cases, which is highly invasive and has a high mortality rate, with the 5-year overall survival (OS) rate being only 6.3%. Age at diagnosis of advanced SCLC is much older, but studies describing the ageing factor for distant metastasis patterns and prognosis of extensive-stage SCLC (ES-SCLC) are limited.

**Methods:** Using the Surveillance, Epidemiology, and End Results (SEER) registry, we identified 18,682 patients with ES-SCLC (9,089 women and 9,053 men) who had complete clinical information between 2008 and 2015. Patients were classified into three groups (older group: ≥80 yrs, middle-aged group: 60-79 yrs, and younger group: ≤59 yrs). The role of different age at diagnosis of ES-SCLC (especially older group) in metastasis patterns was investigated, and OS and cancer-specific survival (CSS) of different age groups of metastatic ES-SCLC was assessed.

**Results:** The most metastasis of ES-SCLC patients in the three groups was multiorgan metastases (MOM) metastasis (71.2%, 70.3% and 66.3%, respectively), the most single organ metastasis in the younger group was the lung (3.3%), the middle-aged group and the older group were the brain (3.5%, 3.1%, respectively). The analysis revealed that older patients were less likely to have MOM, but more likely to have all organs metastases than other two groups (p<0.001). Older group had the worst OS (p<0.001) and CSS (p<0.001). Furthermore, Radiotherapy and chemotherapy can improve survival (p<0.001), but the rate of radiotherapy and chemotherapy in older patients is lower than that in middle-aged and younger patients (50.4% vs 38.6% vs 20.7%, p<0.05). Compared with other two group, older group (odds ratios, ORs) for lung, all organ metastases, and MOM were 0.43 (95% CI 0.27-0.67), 1.77 (95% CI 1.55-2.03), 0.68 (95% CI 0.6-0.77), respectively.

**Conclusion:** The mortality risk is highest with MOM and all organs metastasis followed by brain, lung, bone and liver metastases in elderly ES-SCLC patients. These results will be helpful for pre-treatment evaluation regarding the prognosis of ES-SCLC patients.

## Introduction

Lung cancer remains a serious threat to human health, accounting for 26% of total cancer-related deaths 1. Small cell lung cancer (SCLC) represents about 13% of lung cancer cases, which is highly invasive and has a high mortality rate, with the 5-year overall survival (OS) rate being only 6.3% 2-4. Distant metastasis has occurred in 2/3 of patients with SCLC, and the most common distant metastatic sites included liver, bone and brain, and more than 30% of patients have multiple metastases 5. Studies have shown that age, tumor size, lymph node involvement, behavioral status and gender can all effectively affect SCLC metastases 6.

In recent years, the average age of patients diagnosed with SCLC has increased, with the proportion of SCLC patients over 70 years old increasing from 23% in 1975 to 44% in 2010, and almost half of patients with SCLC are over 70 years old 7,8. Approximately 96.1% of patients with SCLC were over 50 years old when they were diagnosed 9. With the progress of population aging, the number of SCLC patients in the older population will continue to increase. However, in current practice, older patients are always underrepresented in the clinical trials of SCLC and might be undertreated 10.

Due to the limited population-based studies on the practice patterns of older patients with distant metastasis of SCLC and few relevant survival analyses, we performed a retrospective analysis by using the Surveillance, Epidemiology, and End Results (SEER) database, which was aimed at exploring the role of different age groups, especially the elderly, in the diagnosis of SCLC metastasis pattern, and also exploring the survival analyses of SCLC metastasis groups of different ages.

## Methods

### Data source

Using the National Cancer Institute SEER database of (http://seer.cancer.gov/data/options.html), a retrospective analysis was conducted by exempting the data from the institutional review board's oversight. The project of SEER was initiated in 1973 in the USA as population-based registry of cancer which involves about one-tenth of the population in the country. The patient sample of the presented study was selected from the de-identified patients in the NCI SEER 18 Registries (SEER*Stat Database: Incidence-SEER. 18 Custom Data [with additional treatment fields] Nov 2016 Sub), whose data included no personal identifiers and were submitted to the NCI through electronic channels, which are allowed to be used in relevant medical research. The researchers of the present study had obtained the approval from the ethics committee and the institutional review board before using these de-identified data, including the clinicopathological features of the patients, tumor histology, cancer stage, timing and type of the first course treatment, and the therapeutic outcomes. A yearly follow-up rate of 90% for all involved patients whose cancer was diagnosed in recent five years was required for accreditation.

### Study population

This retrospective study assessed the association between MOM and disease-specific and OS in patients with extensive-stage SCLC (ES-SCLC), utilizing data on SCLC patients from the SEER database, maintained by the National Cancer Institute.

**Figure 1:**
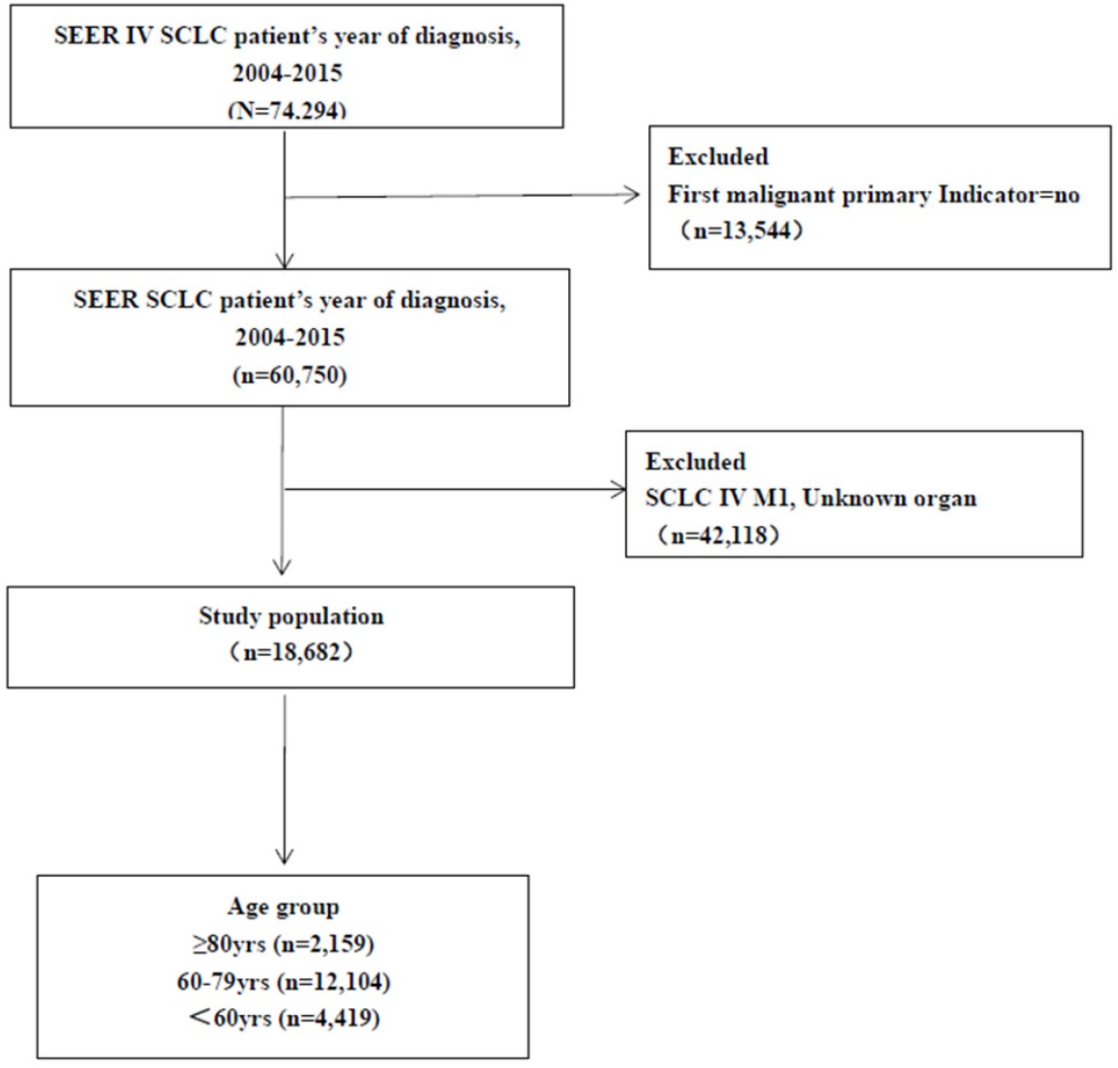
Study flow diagram. Abbreviations: SCLC: small cell lung cancer.

Cases of lung cancer diagnosed from 2008 to 2011 with complete information about distant metastases available in the SEER database were included in this study. Based on imaging studies, distant metastasis refers to the appearance of malignant lesions outside the locoregional thorax and/or mediastinum. Initially, 74,294 cases with SCLC were identified. A total of 18,682 cases were included for analyses, after excluding the following ineligible cases: 13,544 cases with two or more primary sites, 1,817 with incomplete information on organs metastases, 40,301cases with no information on organs metastases (**Figure 1**).

SCLC cases were classified according to histologic type: (1) Site and morphology. Site recoded ICD-O-3/WHO 2008: Lung and Bronchus. (2) International Classification of Diseases for Oncology, 3rd Edition (ICD-03) codes 8002, 8041, 8042, 8043, 8044, and 8045. (3) Patients with American Joint Committee on Cancer (AJCC) stage IV disease. The six groups included patients with (1) bone metastasis without brain, liver, or lung metastasis, (2) brain metastasis without bone, liver, or lung metastasis, (3) liver metastasis without bone, brain, or lung metastasis, (4) lung metastasis without bone, brain, or liver metastases, and (5) two or more metastatic organs among lung, liver, brain and bone (multiorgan metastases, MOM), (6) all metastatic organs with bone, brain, lung and liver metastases. All patients were divided into three groups: ≥80 yrs, 60-79 yrs and ≤59 yrs.

### Statistical analyses

The Pearson Chi square test was used for comparisons of categorical variables. SCLC survival probability was calculated using Kaplan-Meier analysis with follow-up time censored. Cox regression was performed to examine the effect of organ metastasis on disease-specific survival and presented in terms of HRs and 95% CI. A two-tailed *P* value less than 0.05 was considered to be significant. Data were analyzed using the Statistical Package for Social Science version 18.0 (SPSS, Inc., New York, NY, USA).

## Results

### Demographics

We identified 18,682 patients with SCLC metastases from 2008 to 2015, among which 2,159 (11.6%) patients were diagnosed at the age of ≥80 yrs, 12,104 (64.8%) patients 60-79yrs, and 4,419 (23.7%) patients under 59 yrs. The median age at diagnosis was 67yrs, and the mean survival time was 6.9 months. Statistically significant differences of the clinical characteristics among patients in different age groups are summarized in **Table 1**. Specifically, compared to patients in the younger group and the middle-aged group, the older group had a higher rate of the Lower lobe involvement (Lower lobe: 16.8% vs 19.5% vs 25.3%, p<0.001), and lower rate of the lymph node involvement (N3: 26.2% vs 22.6% vs 17.2%, p<0.001). Meanwhile, the older group showed a lower rate of the radiotherapy (50.4% vs 38.6% vs 20.7%, p<0.001) and lower rate of the chemotherapy (79.6% vs 68.3% vs 41.1%, p<0.001).

### Metastasis patterns

**Figure 2:**
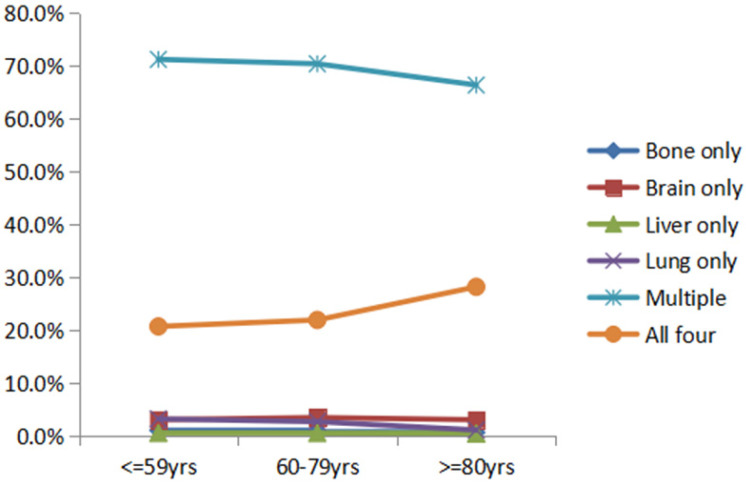
Distant metastatic patterns of different age groups in ES-SCLC patients. Abbreviation: ES-SCLC: extensive stage small cell lung cancer; Metastasis.

ES-SCLC patients with single and multiorgan metastatic disease were analyzed as shown in **Figure 2**. The rates of single-organ metastasis in the younger group, the middle-aged group and the older group were as follows (bone: 1.1% vs 0.9% vs 0.7%, brain: 3.1% vs 3.5% vs 3.1%, liver: 0.6% vs 0.5% vs 0.3%, lung: 3.3% vs 2.7% vs 1.2%, p<0.001). Compared to the younger group and the middle-aged group, the older group had a lower rate of MOM metastasis (71.2% vs 70.3% vs 66.3%, p<0.001), but interestingly, among that the rate of all organs metastasis was higher (20.7% vs 22.0% vs 28.2%, p<0.001).

### Analysis of risk factors associated with different metastasis in ES-SCLC patterns

**Figure 3:**
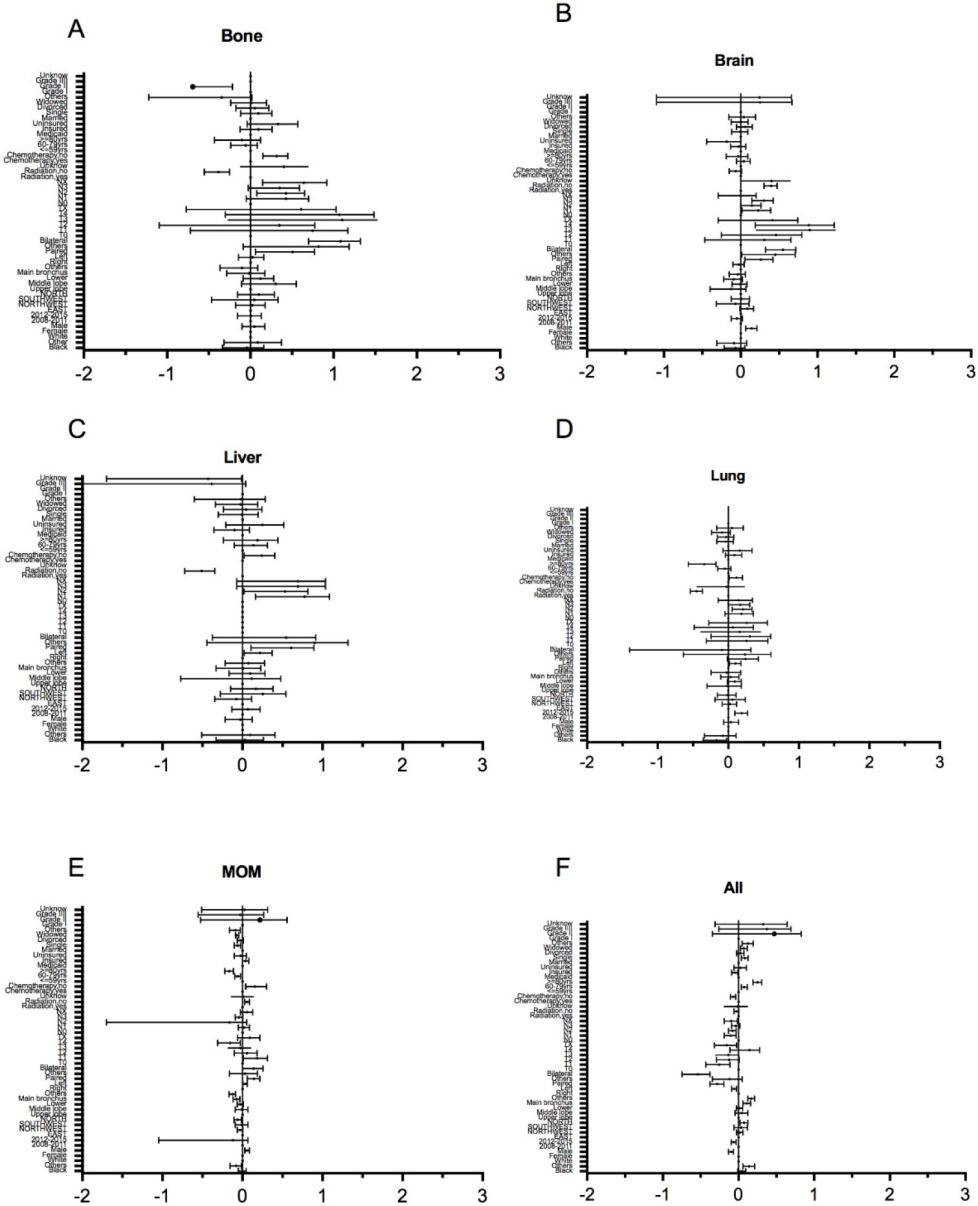
Multivariable logistic regression analyses predicting different sites of metastasis in ES-SCLC patients. (A) only bone metastasis; (B) only brain metastasis; (C) only liver metastasis; (D) only lung metastasis; (E) MOM; (F) all organs metastases.

In logistic regression models adjusted for race, gender, histological grade, T stage, and N stage, the odds for all metastasis in the older group were as follows: all organ metastases were OR: 1.77, 95%CI: 1.55-2.03, and for MOM metastasis were OR: 0.68, 95%CI: 0.6-0.77 and for lung metastasis only were OR: 0.43, 95%CI: 0.27-0.67 (**Figure 3**).

### Analysis of survival outcomes among age groups

**Figure 4:**
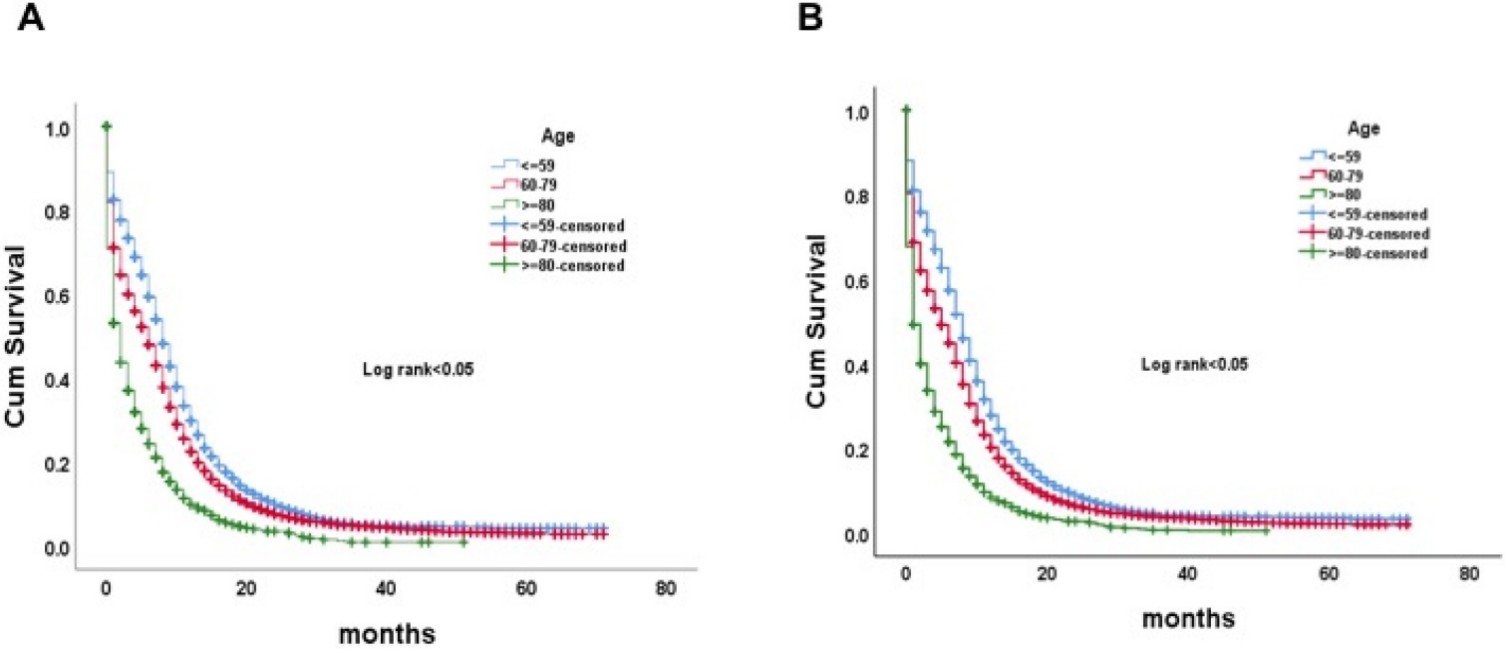
Kaplan-Meier curve of OS (A) and LCSS (B) by age groups among ES-SCLC patients. Abbreviation: OS, overall survival; LCSS, lung cancer-specific survival; ES-SCLC, extensive stage small cell lung cancer.

Overall survival (OS) and lung cancer-specific survival (CSS) were analyzed according to the age groups. The older group had the worst OS and CSS (p <0.05) (**Fig. 4A, B**). The mean OS time was 8.7, 6.8 and 3.6 months respectively in the younger, the middle-aged and the older group.

**Figure 5:**
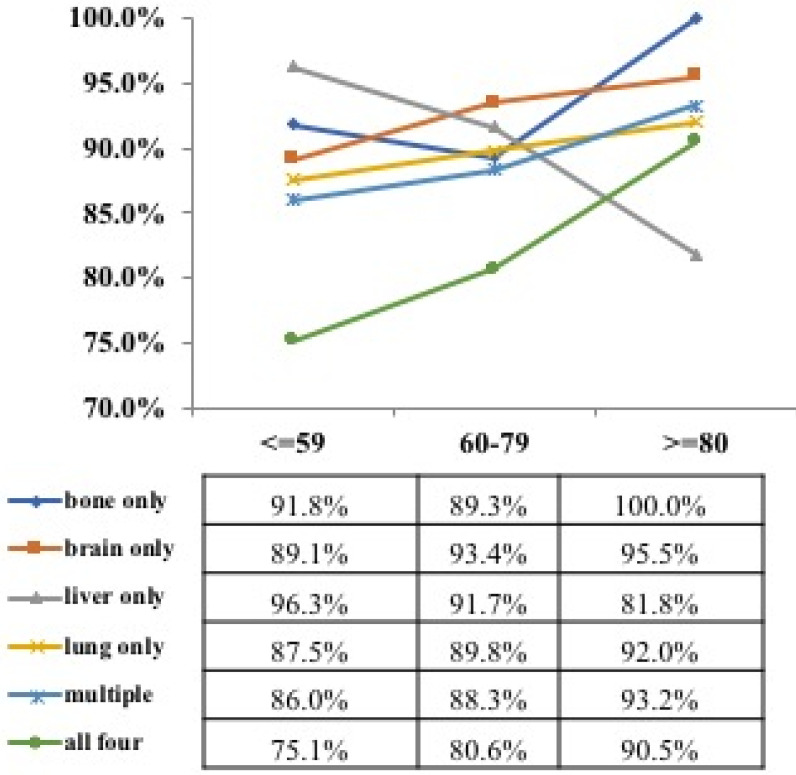
Rates of cancer death for different metastasis sites by age group in ES-SCLC patients.

We analyzed the cancer mortality of different metastatic organs according to the age group. In the older group, the mortality of patients with bone only, brain only, liver only, MOM and all metastasis were as follows (100.0% vs 95.5% vs 81.8% vs 92.0% vs 93.2% vs 90.5%) (**Figure 5**). Furthermore, CSS survival analysis was performed for patients with different organ-specific metastases according to the age group, and all patients in the older group showed poor (as shown in **Supplementary Figure 1A-F**).

Survival analysis of different SCLC organ metastases was as shown in **Supplementary Figure 2**. Bone metastasis only showed the worst OS and CSS in the patients with organ metastases (p<0.05), of which mean survival time was respectively: 3.92 months in OS and 4 months in CCS.

### The influence of radiotherapy and chemotherapy on the survival of patients with SCLC organ metastases

**Figure 6:**
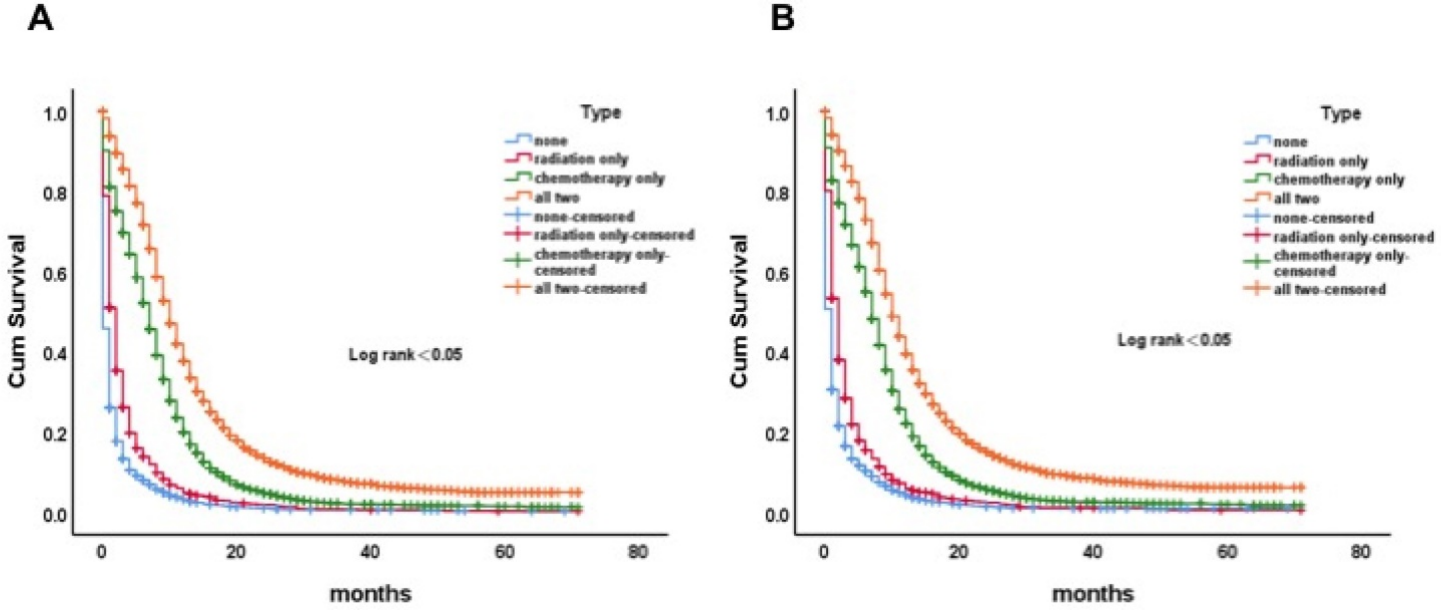
Kaplan-Meier curve of OS (A) and LCSS (B) by additional therapy and without additional therapy in ES-SCLC patients. Abbreviation: OS, overall survival; LCSS, lung cancer-specific survival; ES-SCLC, extensive stage small cell lung cancer.

OS and CSS were analyzed and compared according to the different adjuvant treatment methods. And it showed that radiotherapy and chemotherapy were effective in improving the survival of patients (p<0.05) (**Figure 6A, B**). However, the rates of radiotherapy and chemotherapy in the older group were lower than those in the younger and the middle-aged group (radiotherapy: 50.4% vs 38.6% vs 20.7%, p<0.05, chemotherapy: 79.6% vs 68.3% vs 41.1%, p<0.05).

## Discussion

With the aging process and with the development of diagnosis and treatment technology, the proportion of elderly patients in all cases of SCLC increased from 23% in 1975 to 44% in 2010 11. Different metastasis patterns of SCLC play a different role in the diagnosis 12, especially elderly patients with SCLC have a distinct clinical manifestation and prognosis. In our study, the clinical characteristic parameters showed significant differences among three age groups including race, gender, histologic grades, T stage, N stage, and treatment, and explored the influence of age on the different metastasis patterns. SCLC is the most aggressive form of lung cancer, with rapid cell proliferation and early metastasis, which may be related to the fact that its pathology is a paraneoplastic neuroendocrine lesion 13. At present, it still remains unclear whether the different metastasis patterns and the biological characteristics of tumor are related to the organ defense response at different ages 9. Previous studies have shown that tumor size, lymph node involvement, behavioral status and gender can all effectively affect SCLC metastasis 6, but the analysis of age factor is lacking.

Different from previous studies, there were a total of 18,682 patients with SCLC metastasis in our study, whose median age at diagnosis was 67 years old and their average survival time was 6.9 months. We analyzed the metastasis patterns and survival from the perspective of age: the most metastasis pattern in the three groups of SCLC patients was MOM metastasis, the most single organ metastasis site in the youth group was lung, and in the middle-aged group and the older group was brain, which was consistent with previous literature 14. The incidence of MOM metastasis in the older group was lower than that in the middle-aged group and the younger group, but the incidence of all organs metastasis in the older group was higher than that in the middle-aged group and the younger group. In other words, elderly patients are more likely to have all (four major organs) metastases once they have multiorgan metastases. However, previous studies showed that all (four organ metastases) metastases occurred less in SCLC, and no age grouping was performed 9. Further analysis showed that aging was related to the structural and functional changes of human immune system. Immunosenescence is a progressive decline in immune function and a dynamic process of adaptation 15. The tumor microenvironment of elderly patients has enhanced fibroblast mediated angiogenesis, and the possibility of matrix remodeling and inflammation is greater 16-18. In addition, aging is also related to the increase of gene mutagenesis related to metastasis 19.

Our study also revealed that patients with primary lobe being the upper lobe (45.8% in the young group, 44.8% in the middle-aged group and 44.8% in the older group) accounted for a relatively high proportion, but the lower lobe involvement rate was higher in the older group than in the young group and the middle-aged group (Lower lobe: 16.8% vs 19.5% vs 25.3%; p<0.001). In addition, the involvement rate of lymph nodes in the older group was lower (N3: 26.2%, 22.6%, 17.2%, p<0.001), which may be related to the theory of immunosenescence that is prone to occur in elderly patients 20. At present, this aspect of the research is not very clear, and further experimental verification is needed.

In our Kaplan Meier analysis, as to the survival rates, the older group significantly contributed to the worst OS and the worst CSS (p <0.001) compared with those of the younger group. A variety of factors may help to explain this: reduced physiological reserve in the older group, poor tolerance to cancer treatment, and increased risks of toxicities and death 21,22. In addition, we found that the radiation therapy and chemotherapy would be effective in improving patient survival, but the older group patients were less likely to receive radiation therapy and chemotherapy 23,24. It was also reported that lung cancer patients aged over 80 years old were less likely to receive radiation therapy and chemotherapy as initial treatment than those 70-79 (12.3% vs 40.9%) 25. In spite of some social and economic factors, the effectiveness of the radiation therapy and chemotherapy on patients in older group does exist apparently and we should enhance the survival benefit actively from radiation therapy and chemotherapy.

Our research also has certain limitations. Firstly, no information was provided in the SEER database such as physical state (PS) assessment of cancer patients, specific therapeutic method, gene mutation type in patients with lung cancer, whether to conduct targeted therapy and immune therapy, and etc., which may influence the prognosis significantly. In addition, our study failed to further elucidate the specific organs where the tumor has metastasized in the multiorgan metastases cohort.

In conclusion, based on a large sample of the population in the SEER , our study analyzed the influence of aging factors on metastasis patterns of SCLC patients, and summarized that the incidence of MOM metastasis in the older group was lower than that in the middle-aged group and the young group, but the incidence of all-metastasis in the older group was higher than that other two groups, suggesting that aging is an important factor affecting the metastasis patterns, and age-related tumor biology and genetics should be further explored. In addition, the study also showed that the OS rate of elderly patients was low, and local treatments such as radiotherapy and chemotherapy accounted for a relatively low proportion. We hope to provide more comprehensive treatment for elderly patients.

## Supplementary Material

Supplementary figures.

## Figures and Tables

**Table 1:** Characteristics of patients with distant metastatic SCLC by age groups

Characteristics	Age			Total	χ^2^	P
	≤59	60-79	≥80			
**Race (n, %)**					60.219	0.000
White	3773 (85.4)	10544 (87.1)	1885 (87.3)	16202 (86.7)		
Black	502 (11.4)	1076 (8.9)	149 (6.9)	1727 (9.2)		
Others	144 (3.3)	484 (4)	125 (5.8)	753 (4)		
**Sex**					8.047	0.018
Female	2102 (47.6)	5880 (48.6)	1107 (51.3)	9089 (48.7)		
Male	2317 (52.4)	6224 (51.4)	1052 (48.7)	9593 (51.3)		
**Year of diagnosis**					10.086	0.006
2008-2011	1516 (34.3)	3845 (31.8)	718 (33.3)	6079 (32.5)		
2012-2015	2903 (65.7)	8259 (68.2)	1441 (66.7)	12603 (67.5)		
**Region**					113.868	0.000
EAST	2400 (54.3)	6226 (51.4)	943 (43.7)	9569 (51.2)		
NORTHWEST	1219 (27.6)	3971 (32.8)	847 (39.2)	6037 (32.3)		
SOUTHWEST	153 (3.5)	416 (3.4)	61 (2.8)	630 (3.4)		
NORTH	647 (14.6)	1491 (12.3)	308 (14.3)	2446 (13.1)		
**Primary labeled**					144.424	0.000
Upper lobe, lung	2025 (45.8)	5418 (44.8)	794 (36.8)	8237 (44.1)		
Middle lobe, lung	134 (3)	425 (3.5)	84 (3.9)	643 (3.4)		
Lower	743 (16.8)	2364 (19.5)	546 (25.3)	3653 (19.6)		
Main bronchus	596 (13.5)	1366 (11.3)	178 (8.2)	2140 (11.5)		
Others	921 (20.8)	2531 (20.9)	557 (25.8)	4009 (21.5)		
**Grade**					7.303	0.294
I	4 (0.1)	17 (0.1)	2 (0.1)	23 (0.1)		
II	4 (0.1)	28 (0.2)	2 (0.1)	34 (0.2)		
III	330 (7.5)	977 (8.1)	166 (7.7)	1473 (7.9)		
Unknown	4081 (92.4)	11082 (91.6)	1989 (92.1)	17152 (91.8)		
**Laterality**					13.276	0.103
Right	2304 (52.1)	6543 (54.1)	1172 (54.3)	10019 (53.6)		
Left	1810 (41)	4753 (39.3)	816 (37.8)	7379 (39.5)		
Paired	221 (5)	593 (4.9)	120 (5.6)	934 (5)		
Others	33 (0.7)	71 (0.6)	15 (0.7)	119 (0.6)		
Bilateral	51 (1.2)	144 (1.2)	36 (1.7)	231 (1.2)		
**T Stage**					63.011	0.000
T0	50 (1.1)	151 (1.2)	23 (1.1)	224 (1.2)		
T1	274 (6.2)	905 (7.5)	143 (6.6)	1322 (7.1)		
T2	839 (19)	2522 (20.8)	494 (22.9)	3855 (20.6)		
T3	839 (19)	2281 (18.8)	424 (19.6)	3544 (19)		
T4	1847 (41.8)	4539 (37.5)	721 (33.4)	7107 (38)		
TX	570 (12.9)	1706 (14.1)	354 (16.4)	2630 (14.1)		
**AJCC**					111.836	0.000
N0	500 (11.3)	1461 (12.1)	341 (15.8)	2302 (12.3)		
N1	249 (5.6)	759 (6.3)	131 (6.1)	1139 (6.1)		
N2	2333 (52.8)	6625 (54.7)	1165 (54)	10123 (54.2)		
N3	1156 (26.2)	2737 (22.6)	372 (17.2)	4265 (22.8)		
NX	181 (4.1)	522 (4.3)	150 (6.9)	853 (4.6)		
**Radiation**					562.762	0.000
Yes	2225 (50.4)	4673 (38.6)	447 (20.7)	7345 (39.3)		
No	2140 (48.4)	7303 (60.3)	1701 (78.8)	11144 (59.7)		
Unknown	54 (1.2)	128 (1.1)	11 (0.5)	193 (1)		
**Chemotherapy**					987.223	0.000
Yes	3516 (79.6)	8263 (68.3)	887 (41.1)	12666 (67.8)		
No	903 (20.4)	3841 (31.7)	1272 (58.9)	6016 (32.2)		
**Insurance Recode**					1425.684	0.000
Medicaid	1327 (30)	1661 (13.7)	184 (8.5)	3172 (17)		
Insured	2650 (60)	10147 (83.8)	1965 (91)	14762 (79)		
Uninsured	442 (10)	296 (2.4)	10 (0.5)	748 (4)		
**Marital status**					1726.94	0.000
Married	2001 (45.3)	6160 (50.9)	884 (40.9)	9045 (48.4)		
Single	1207 (27.3)	1676 (13.8)	159 (7.4)	3042 (16.3)		
Divorced	805 (18.2)	1733 (14.3)	168 (7.8)	2706 (14.5)		
Widowed	189 (4.3)	2035 (16.8)	848 (39.3)	3072 (16.4)		
Others	217 (4.9)	500 (4.1)	100 (4.6)	817 (4.4)		
**Education**					20.299	0.002
≥21	932 (21.1)	2465 (20.4)	383 (17.7)	3780 (20.2)		
13-21	1351 (30.6)	3768 (31.1)	661 (30.6)	5780 (30.9)		
7-12.99	1861 (42.1)	5148 (42.5)	945 (43.8)	7954 (42.6)		
<7	275 (6.2)	723 (6)	170 (7.9)	1168 (6.3)		
**Median income level**					143.457	0.000
<38,000	556 (12.6)	1221 (10.1)	120 (5.6)	1897 (10.2)		
38,000-47,999	923 (20.9)	2467 (20.4)	359 (16.6)	3749 (20.1)		
48,000-62,999	1729 (39.1)	4804 (39.7)	855 (39.6)	7388 (39.5)		
>63000	1211 (27.4)	3612 (29.8)	825 (38.2)	5648 (30.2)		

## Abbreviations

SCLC: small cell lung cancer
OS: overall survival
LCSS: lung cancer specific survival
SEER: Surveillance, Epidemiology, and End Results registry
OR: odds ratio
CI: confidence interval
NOS: not otherwise specified
ES-SCLC: extensive stage small cell lung cancer
MOM: multiorgan metastatic
