# Supplementary material for: Ageing as key factor for distant metastasis patterns and prognosis in patients with extensive-stage Small Cell Lung Cancer
Source: J Cancer. 2021 Jan 15;12(6):1575–82. doi: 10.7150/jca.49681 (PMC7890308; doi:10.7150/jca.49681)

Figure S1. LCSS by age groups among ES-SCLC in different sites of metastasis. (A) only bone metastasis; **(B)** only brain metastasis; **(C)** only liver metastasis; **(D)** only lung metastasis; **(E)** MOM; **(F)** all organs metastases. Abbreviation: LCSS, lung cancer-specific survival; MOM, multiorgan metastatic; ES-SCLC, extensive stage small cell lung cancer

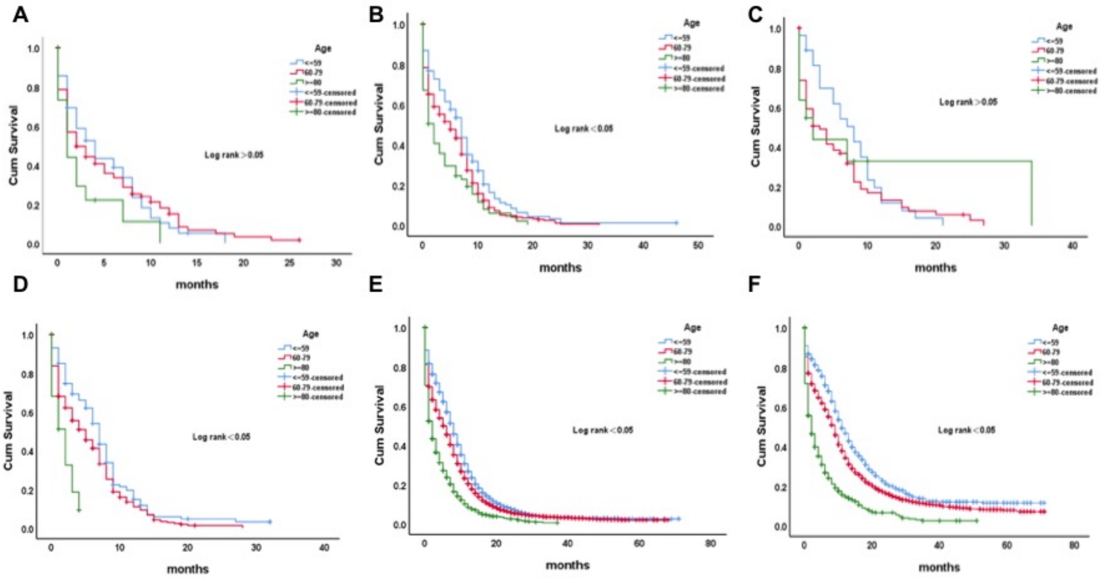

Figure S2. Kaplan-Meier curve of OS (A) and LCSS (B) by different sites of metastasis among ES-SCLC. Abbreviation: OS, overall survival; LCSS, lung cancer-specific survival; ES-SCLC, extensive stage small cell lung cancer

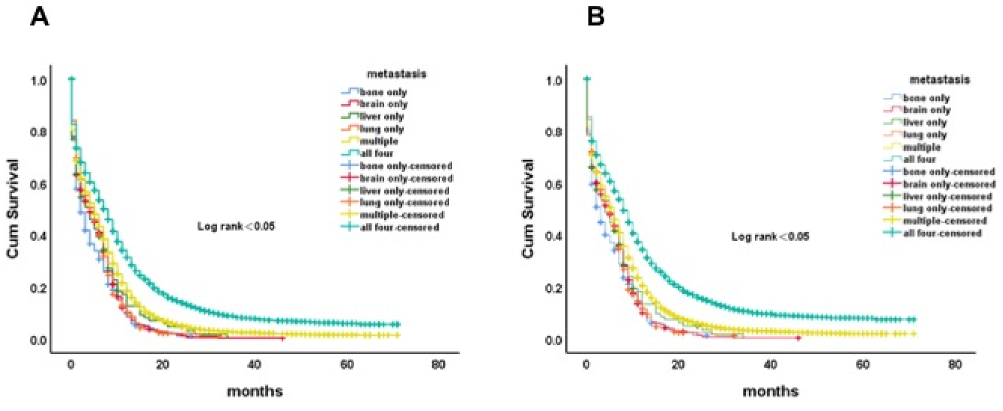

Supplement: Supplementary file 1 — Supplementary figures. [file jcav12p1575s1.pdf]
